# Bicyclomycin Activity against Multidrug-Resistant Gram-Negative Pathogens

**DOI:** 10.1128/spectrum.03790-22

**Published:** 2022-12-19

**Authors:** Weijie Wang, Xiaoli Zhu, Huan Luo, Zheng Wang, Anjin Hong, Jie Zeng, Li Li, Dai Wang, Xianming Deng, Xilin Zhao

**Affiliations:** a State Key Laboratory of Molecular Vaccinology and Molecular Diagnostics, Department of Laboratory Medicine, School of Public Health, Xiamen University, Xiamen, Fujian, China; b State Key Laboratory of Cellular Stress Biology, Innovation Center for Cell Signaling Network, School of Life Sciences, Xiamen University, Xiamen, Fujian, China; c State-Province Joint Engineering Laboratory of Targeted Drugs from Natural Products, Xiamen University, Xiamen, Fujian, China; Indian Institute of Science Bangalore

**Keywords:** bicyclomycin, multidrug-resistant Gram-negative bacteria, murine model of infection, MIC distribution, lethal synergy

## Abstract

The growing prevalence of antimicrobial resistance poses a grave threat to human health. Among the most difficult bacterial infections to treat are those caused by multidrug-resistant (MDR) Gram-negative pathogens because few effective regimens are available. One approach to this problem is to find ways to increase the activity of old antimicrobials that had seen limited application. Bicyclomycin, an inhibitor of transcription termination, is an example in which the additional inhibition of protein or RNA synthesis increases bicyclomycin-mediated lethality against Gram-negative bacteria. To examine the potential of bicyclomycin for the treatment of MDR bacterial pathogens, we first measured the MICs of bicyclomycin and other widely used antimicrobials against more than 100 multidrug-resistant Gram-negative clinical isolates. Bicyclomycin showed good coverage of carbapenem-resistant *Enterobacteriaceae* (CRE) and Escherichia coli (MIC_50_/MIC_90_ of 25/50 μg/mL for both bacteria) and moderate activity against Klebsiella pneumoniae (MIC_50_/MIC_90_ of 50/200 μg/mL). Bicyclomycin also exhibited synergy (e.g., fractional inhibitory concentration [FIC] index of <0.5) with doxycycline for the inhibition of bacterial growth by a checkerboard assay. Although bicyclomycin exhibited very weak lethality by itself, it showed synthetic lethality with doxycycline against K. pneumoniae: the combination killed 100- to 1,000-fold more bacteria than either agent alone. In a murine model of infection, the bicyclomycin-doxycycline combination showed better efficacy than either agent alone, and the combination treatment largely eliminated histopathological manifestations caused by infection. Thus, bicyclomycin, which has largely been limited to the treatment of Gram-negative digestive tract infections, can now be considered for the combination treatment of systemic multidrug-resistant infections caused by CRE, E. coli, and K. pneumoniae.

**IMPORTANCE** As antimicrobial resistance continues to increase, options for effectively treating multidrug-resistant (MDR) Gram-negative infections are declining. Finding ways to enhance the lethality of old agents that have unique molecular targets is important because developing new antimicrobials is becoming increasingly difficult. The present work showed that the old antibiotic bicyclomycin has good bacteriostatic activity against multiple clinical isolates of three significant types of MDR Gram-negative pathogens frequently encountered in hospital infections, as required for the consideration of expanded indications. More significant is the synergistic growth-inhibitory effect and the enhancement of killing by the additional presence of doxycycline since this increases the *in vivo* efficacy. These data demonstrate that bicyclomycin-containing regimens have potential as new treatment options for MDR Gram-negative infections such as those caused by CRE, E. coli, and K. pneumoniae.

## OBSERVATION

The prevalence of antimicrobial resistance has reached a point where the need for novel agents and/or new treatment regimens is critical. This is particularly true for multidrug-resistant (MDR) Gram-negative pathogens (1, 2) such as carbapenem-resistant *Enterobacteriaceae* (CRE). Since the development of new antimicrobials has encountered a bottleneck (2), reviving/repurposing old drugs has become an attractive alternative. Daptomycin and retapamulin have served as successful examples with Gram-positive pathogens (3, 4). For Gram-negative bacteria, we have been examining bicyclomycin, an old agent whose use was restricted by poor oral absorption and weak bactericidal activity (5–8). However, bicyclomycin has good pharmacokinetic profiles following intramuscular or intravenous administration (7), making it feasible for the treatment of MDR Gram-negative infections encountered in hospitals where systemic drug administration is routine.

Progress with the weak-lethality problem occurred when we discovered that combining bicyclomycin with a bacteriostatic inhibitor of protein or RNA synthesis converts bicyclomycin from a largely bacteriostatic drug to an active bactericidal agent (6). Whether bicyclomycin has *in vitro* activity against a large panel of clinical isolates and whether its synthetic lethality occurs in animal models of infection remain unknown.

**FIG 1:**
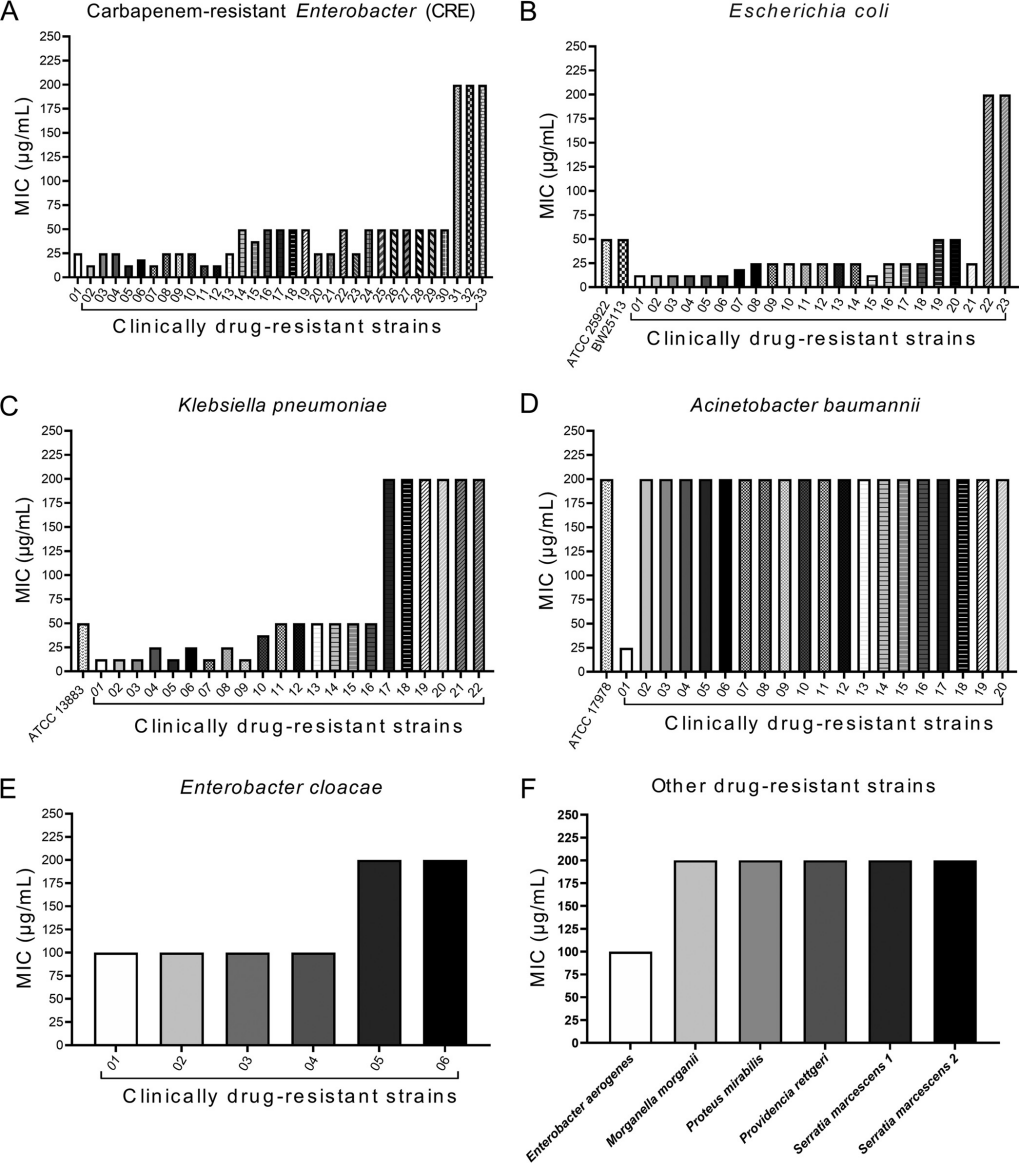
Bicyclomycin MIC distributions of clinically drug-resistant bacterial strains. (A) CRE clinical isolates (*n* = 33). (B) E. coli ATCC 25922 and BW25113 reference strains along with 23 clinical isolates. (C) K. pneumoniae ATCC 13883 reference strain along with 22 clinical isolates. (D) A. baumannii ATCC 17978 reference strain along with 20 clinical isolates. (E) Enterobacter cloacae clinical isolates (*n* = 6). (F) Other drug-resistant clinical isolates (as listed on the *x* axis) tested (*n* = 6). The *x* axis lists strain numbers for each bacterial species tested, except for panel F, which lists bacterial species. Similar results were obtained in a replicate experiment.

When we examined 110 MDR clinical isolates, along with 4 reference strains, for MICs, most strains were resistant to β-lactams and other commonly used antimicrobials (see Table S1 in the supplemental material). These bacteria were categorized into 4 different specific Gram-negative bacterial species (Klebsiella pneumoniae [*n* = 22], Escherichia coli [*n* = 23], Acinetobacter baumannii [*n* = 20], and Enterobacter cloacae [*n* = 6]) that are susceptible to carbapenems (e.g., MIC of meropenem of <4 μg/mL); a category called other drug-resistant strains (*n* = 6), which includes different Gram-negative bacterial species other than the 4 bacterial species listed above; and a category of various *Enterobacteriaceae* species (*n* = 33) that are resistant to carbapenem (CRE). The bicyclomycin MIC ranged between 6.25 and 200 μg/mL, with MIC_50_ and MIC_90_ values being 25 μg/mL and 50 μg/mL, respectively, for both CRE and E. coli; these values were 50 μg/mL and 200 μg/mL, respectively, for K. pneumoniae (Table S1). Both the MIC_50_ and the MIC_90_ were 200 μg/mL for A. baumannii and other bacterial species tested; these values were 100 μg/mL and 200 μg/mL, respectively, for E. cloacae (Table S1). The distribution of bicyclomycin MIC values among the six MDR bacterial species/categories is shown in Fig. 1, with the following MIC-based susceptibility ranking order (from high to low): CRE = E. coli > K. pneumoniae > E. cloacae > A. baumannii = other drug-resistant bacterial species tested. Bicyclomycin also exhibited a synergistic effect with doxycycline for the combinational inhibition of bacterial growth when isolates susceptible to both compounds were tested in a checkerboard assay, resulting in average fractional inhibitory concentration (FIC) indices being slightly below 0.5 (Table S2). These data suggest that bicyclomycin might effectively treat MDR infections caused by CRE, E. coli, and many K. pneumoniae strains; effective treatment is less likely for infections caused by A. baumannii, E. cloacae, or other bacterial species tested.

**FIG 2:**
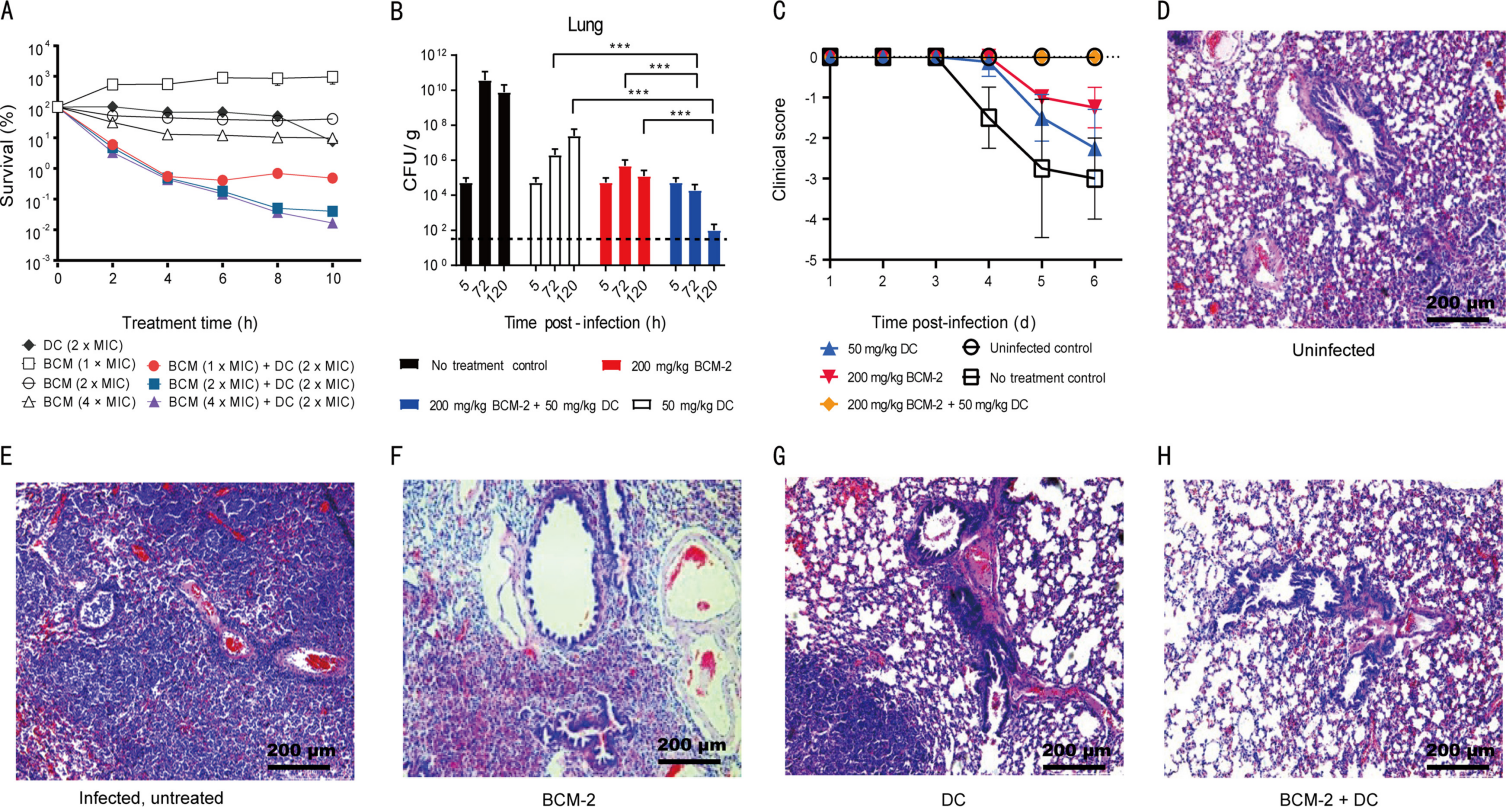
Synergistic efficacy of the bicyclomycin-doxycycline combination against K. pneumoniae. (A) Synergistic lethality of bicyclomycin with doxycycline *in vitro*. Exponentially growing K. pneumoniae 41053 cells were treated with 2× MIC of doxycycline (DC) or the indicated concentrations of bicyclomycin (BCM) alone or with various combinations of bicyclomycin plus doxycycline for the indicated times. The MICs of bicyclomycin and doxycycline were 20 and 4 μg/mL, respectively. The experiments were done in triplicate; error bars indicate standard deviations (SD). (B) Synergistic lethality of bicyclomycin with doxycycline in a murine model of infection. Mice infected with K. pneumoniae strain 41053 were untreated or treated with 5% (wt/vol) Kolliphor HS 15 (no-treatment control), doxycycline alone, bicyclomycin benzoate (BCM-2) alone, or doxycycline plus bicyclomycin benzoate at the indicated concentrations once daily starting at 5 h postinfection. The bacterial burden in the lungs was assessed at the indicated times. Each treatment group included 8 mice, and each time point included samples taken from 4 mice. Error bars indicate means ± SD. The dotted lines indicate the detection limit (33 CFU/g). ***, *P < *0.001. (C) A behavioral evaluation was performed for infected mice at the indicated times postinfection. The clinical scores were obtained by observing posture, paralysis, tremor, body weight, fur, and body temperature. Scores are as follows: −5 for death, −4 for dying, −3 for severe illness, −2 for sickness, −1 for mild symptoms, and 0 for asymptomatic. (D to H) Mitigation of lung histopathology by bicyclomycin, doxycycline, and their combination. Representative histological results (hematoxylin-eosin staining) for lungs collected at 5 days postinfection are shown for uninfected mice (D), infected untreated mice (5% [wt/vol] Kolliphor HS 15 solvent control) (E), and infected mice treated 200 mg/kg bicyclomycin benzoate (F), 50 mg/kg doxycycline (G), and 200 mg/kg bicyclomycin benzoate plus 50 mg/kg doxycycline (H). Similar results were obtained in 2 replicate experiments.

Since combining bicyclomycin with an inhibitor of transcription or translation can confer lethal synergy (synthetic lethality) (6), we asked whether lethal synergy applies to K. pneumoniae, a bacterium frequently encountered in carbapenem-resistant, nosocomial outbreaks (9, 10). The MICs of bicyclomycin and doxycycline were 20 and 4 μg/mL, respectively, for the clinical strain 41053. The combination of bicyclomycin and doxycycline killed >100-fold more bacteria than either agent alone at concentrations slightly above the MIC for both drugs (Fig. 2A). Similar synthetic lethality was observed for three other independent isolates (Fig. S1).

To determine whether the bicyclomycin-doxycycline combination also confers lethal synergy *in vivo*, we examined lung infections caused by the highly virulent K. pneumoniae strain 41053 in immunocompetent mice. When mice were inoculated with 5 × 10^3^ CFU of bacteria, the lung bacterial burden increased up to 4 days postinfection (Fig. S2A), and the clinical scores gradually decreased (Fig. S2B); no animal death occurred until day 5 postinfection. When uninfected animals were treated with bicyclomycin (200 mg/kg of body weight), doxycycline (50 mg/kg), or both in combination, little toxicity was observed, as indicated by the lack of weight loss (Fig. S3). Treatment reduced the bacterial burdens in the lung (Fig. 2B), heart, liver, spleen, and kidney (Fig. S4A to D) by 10- to 10,000,000-fold, depending on the organ, the drug used, and the treatment time. Moreover, it improved the clinical scores of infected animals, with the combination treatment showing the best efficacy (Fig. 2C).

Pathophysiological changes in the lung revealed by hematoxylin-eosin staining showed that uninfected, control mice exhibited no granulocyte infiltration or other inflammatory signs; alveolar septa and blood vessels were intact (Fig. 2D). Infected, untreated mice exhibited hemorrhage, the formation of interstitial edema in alveolar septa, and the infiltration of the perivascular space by erythrocytes and granulocytes (Fig. 2E). Only slight hemorrhaging was seen in the bicyclomycin- or doxycycline-treated groups, although the formation of interstitial edema plus erythrocyte and granulocyte infiltration were evident (Fig. 2F and G). The bicyclomycin-doxycycline combination group showed neither granulocyte infiltration nor other inflammatory signs, and the lung alveolar structure was intact (Fig. 2H). These data indicate that bicyclomycin, especially when combined with doxycycline, can both drastically reduce the bacterial burden and mitigate/eliminate pathological symptoms in the infected lung.

Bicyclomycin is an attractive agent for MDR Gram-negative infections due to previous work (8, 11–13), our finding of activity against many isolates, and our demonstration of synthetic lethality in a murine infection by K. pneumoniae. This agent has a unique mechanism of action (blocking Rho-dependent transcription termination), which makes cross-resistance to other compounds unlikely (12, 14). Moreover, bicyclomycin is rarely used clinically, which has limited the emergence of resistance. These observations, plus a good safety profile (5, 12), encourage the further development of bicyclomycin-protein/RNA synthesis inhibitor combinations as therapies for MDR Gram-negative infections, particularly those caused by carbapenem-resistant *Enterobacteriaceae*, extended-spectrum-β-lactamase (ESBL)-producing E. coli, and MDR K. pneumoniae. The availability of a wide variety of protein/RNA synthesis inhibitors that can be combined with bicyclomycin for synergistic killing partially mitigates resistance problems with combination therapy because the chances of an isolate being resistant to all protein and RNA synthesis inhibitors are low. The poor oral absorption of bicyclomycin (7, 8, 15, 16) can be readily bypassed by systemic administration (7).

Clinical isolates were obtained from the Second Affiliated Hospital of Fujian Medical University (Fujian, China) and the Centers for Disease Control and Prevention Antibiotic Resistance Isolate Bank (Atlanta, GA, USA). Escherichia coli ATCC 25922, E. coli BW25113, Klebsiella pneumoniae ATCC 13883, and Acinetobacter baumannii ATCC 17978 were included as reference strains.

Bicyclomycin, obtained from Andrew Truman (John Innes Center, UK), was dissolved in sterile water. Bicyclomycin benzoate was purchased from BioAustralis Fine Chemicals Company (NSW, Australia); it was dissolved in 5% (wt/vol) Kolliphor HS 15 (catalog number 42966; Sigma-Aldrich, St. Louis, MO, USA). Doxycycline hydrochloride was purchased from Sangon Biotech Company (Shanghai, China); it was dissolved in 0.9% NaCl. Other antimicrobials were obtained from bioMérieux (Lyon, Rhône, France).

Bacterial strains were grown at 37°C in Luria-Bertani (LB) or brain heart infusion (BHI) broth or on LB or BHI agar purchased from Becton, Dickinson and Company (Franklin Lakes, NJ, USA). The MIC of bicyclomycin was determined using staggered, 2-fold broth dilution according to Clinical and Laboratory Standards Institute guidelines (17). The MICs of other antimicrobials were determined using the bioMérieux Vitek 2 automated system. Bacterial killing assays were performed by treating exponentially growing cultures (5 × 10^8^ CFU/mL) with drugs, followed by diluting, plating, and incubating the cultures on drug-free agar at 37°C for colony enumeration. For killing using a combination of doxycycline and bicyclomycin, doxycycline was added 30 min before bicyclomycin was added.

For murine lung infection, female ICR mice (6 to 8 weeks old), obtained from the Xiamen University Experimental Animal Center, were maintained at a controlled temperature with free access to food and water. Mice were infected with ~5 × 10^3^ CFU of K. pneumoniae 41053 (Anhui Medical University) using a mouse intubation pack (catalog number RW-A3747; Braintree Scientific, Inc., Braintree, MA). Mice were injected intraperitoneally with 1.25% 2,2,2-tribromoethanol (25 mg/kg) for anesthesia, and the glottis of each mouse was then opened. An intravenous catheter (B. Braun Introcan, catalog number 4254090B; B. Braun Melsungen AG, Germany) was inserted into the mouse windpipe to deliver 10 μL of the bacterial suspension to the bronchus. Bacteria traveled to the lungs and established systemic infection. Once-daily antimicrobial administration was initiated at 5 h postinfection by oral gavage (doxycycline) or intraperitoneal injection (bicyclomycin benzoate). Animals were observed daily for survival and the clinical score, a matrix of animal behavior parameters (posture, paralysis, tremor, body weight, fur, and body temperature, etc.). The bacterial burden was measured by homogenizing the organs after the animals were euthanized and plating diluted samples onto agar for bacterial enumeration. Infected lungs were also stained with hematoxylin-eosin for histopathological analysis (18). Animal protocol XMULAC201900295 was approved by the institutional animal care and use committee.
